# The reliability and validity of the training elements scale for clinicians in the new era——based on the perspective of Chinese doctors’ job demands

**DOI:** 10.1186/s12909-023-04289-y

**Published:** 2023-05-02

**Authors:** Weiqiong Jin, Yinghui Fang, Yuchen Zhang, Yijun Lv

**Affiliations:** 1 Academic Affairs Office, Wenzhou Medical University, Wenzhou, 325000 China; 2 Key Research Center of Philosophy and Social Sciences of Zhejiang Province (Institute of Medical Humanities), Wenzhou Medical University, Wenzhou, 325000 China; 3 Alberta Institute, Wenzhou Medical University, Wenzhou, 325000 China; 4 Party Committee, Wenzhou Medical University, Wenzhou, 325000 China; 5 Wenzhou Medical University, Ouhai District, Tongxin Building, Chashan Higher Education Park, Zhejiang Wenzhou, China

**Keywords:** Clinician, Talents training, Required elements, Reliability, Validity

## Abstract

**Objective:**

The purpose of this study was to develop a scale of clinician training elements in the new period and test its reliability and validity.

**Methods:**

Our approach was based on interdisciplinary theory, systematology, collaborative innovation theory, and whole person education theory combined with the existing post competency model of Chinese doctors and the responsibilities and requirements endowed clinicians in the new historical period. The scale elements were extracted by referring to the relevant literature, and the training elements scale for clinicians in the new period were preliminarily formed. From July to August 2022, 1,086 clinicians from tertiary medical institutions in eastern, central, and western China were sampled and investigated. The questionnaire was revised via the critical ratio method and homogeneity test method, and the reliability and validity of the scale were also tested.

**Results:**

The training elements scale for clinicians in the new period included the following eight dimensions: basic clinical knowledge, interdisciplinary knowledge, clinical skill operation, public health knowledge, technological innovation capability, lifelong learning needs, medical humanistic literacy, and international exchange vision, as well as 51 other items. The Cronbach’s α coefficient of the scale was 0.981, the half-reliability was 0.903, and the average variance extraction of each dimension was greater than 0.5. An exploratory factor analysis extracted eight main factors, and the cumulative variance contribution rate was 78.524%. Confirmatory factor analysis showed that the model fit was ideal and the factor structure was stable.

**Conclusion:**

The clinician training factor scale in the new era can fully meet the current training needs of r clinicians, and has good reliability and validity. It can be widely used in medical colleges and universities as a reference to reform the content of medical training and education, and can also be used in the continuing education of clinicians after graduation to compensate for gaps in knowledge during clinical work.

## Background

With the national health policy change in China, and the impact of public health emergencies on the medical and health industries, colleges and universities were required to continuously adjust talent training programmes and cultivate clinicians who met the needs of clinical positions. The construction objectives and tasks of ‘Healthy China’ were first proposed at the Health and Wellness Conference held in China in 2016. The main thrust of the Chinese healthcare system was to achieve the transformation from treating diseases as the core of healthcare to providing health and wellness services throughout a person’s life cycle [1]. Accordingly, this required colleges and universities to cultivate medical talents, not only to teach them to treat patients, but also to allow them to master the knowledge of prevention and provide full-cycle services for their patients’ health. In October 2018, to implement the spirit of national policy documents, the Ministry of Education of China, the National Health and Health Commission, and the State Administration of Traditional Chinese Medicine issued ‘The Opinions on Strengthening the Collaboration between Medicine and Education to Implement the Excellent Doctor Education and Training Program 2.0’. This was a proposal to promote competency-oriented education and teaching reformation, thereby focusing on improving the professional literacy and clinical practice ability of medical students [2]. At the beginning of 2020, the coronavirus disease 2019 (COVID-19) pandemic spread, and the medical industry faced huge pressures and challenges. While doctors remained at their posts, they experienced problems in their work, such as lack of public health knowledge [3], interruption of health care services [4], insufficient self-protection ability [5], and decreased mental capacity [6].

In order to cultivate medical talents adapted to these needs, in July 2020, the Chinese government issued the ‘Guiding Opinions on Accelerating the Innovative Development of Medical Education’, which clearly stated that, to strengthen general practice and public health personnel training, it would be necessary to reinforce the knowledge integration of medical + X multidisciplinary background in the process of clinical medical personnel training [7], where X could be science, engineering, liberal arts, etc. Other countries also adjusted their medical talent training models in the context of the COVID-19 pandemic. The United States guided medical students to use virtual technology more and learn telemedicine and its related treatment methods [8]. Singapore strengthened its protection training of medical students for infectious diseases and paid attention to the cultivation of students’ clinical core abilities [9]. In Australia, they hoped to cultivate the ability of medical students to solve clinically complex problems, as well as the ability to form functional groups between hospitals and communities to improve the post level of future doctors [10]. Medical professionals in Germany believed that medical education had entered a period of digital transformation. Given this, medical students in Germany were allowed to master digital technology in advance before integrating into the healthcare system to improve their ability to cope with public emergencies [11]. However, it is evident from the current research literature that there are still limitations regarding certain aspects of the research field and the corresponding training programme adjustments. Moreover, no relevant discussion has been yet conducted regarding the comprehensive investigation and analysis of the training elements required by medical students. Although the Chinese government offered macroscopic guidance in their education policies, specific implementation rules are still lacking. Therefore, this study hoped to construct a set of clinician training elements from the perspective of medical post demands in the modern context through the investigation of clinicians in medical institutions, in order to provide guidance to medical colleges on how to conduct medical personnel training.

## Material and methods

### Theoretical basis for scale development

This study was based on interdisciplinary theory, systematology, collaborative innovation theory, and the theory of holistic education, combined with the policy document spirit of medical education reform in China. This research investigated the new needs of clinicians’ posts in the post-pandemic period. Interdisciplinary theory suggests that, in the fields of human health, scientific research, and sustainable social development, a single subject knowledge has been unable to solve these types of complex topics [12]; as such, medical posts continued to face complex and comprehensive problems in clinical practice, and it was, therefore, necessary to strengthen multidisciplinary horizontal linkage [13]. This was especially the case concerning the blind area exposure of clinicians to interdisciplinary knowledge of medicine, engineering, arts and science, response to public emergencies, science and technology, and other subject knowledge after the COVID-19 pandemic. Hence, it was necessary to understand their actual needs for theoretical knowledge, public health knowledge, scientific and technological innovation knowledge, and other contents involving medical interdisciplinary content.

The core of system theory resembles that of interdisciplinary theory. Indeed, the former holds that medical education should be regarded as a comprehensive system composed of multiple subsystems and elements, rather than an isolated discipline. The subsystem comprises professional theoretical education, medical practice education, and medical humanistic education, among others; additionally, its elements include, but are not limited to, personnel training objectives, curriculum, and organisational management. The internal elements of the system interact with each other and are influenced by the external environment. The role and function of the system could only be better exerted by analysing the composition of the medical education system at multiple levels, combining it with the needs of clinical posts, and continuously optimising the system structure [14].

Based on systematology, collaborative innovation theory defined innovation as a new system, and the medical education system achieved the common goal of innovation through the internal elements and the synergistic effect between the system and the environment in which it was located [15]. The overall goal of the medical education system is to coordinate and unify—that is, to cultivate medical talents in the new era to meet current economic and social needs—but collaborative innovation theory suggests that the objectives of each subsystem may be different, and investigation of professional, practical, and humanistic education, and other subsystems from the internal horizontal was helpful to build a more comprehensive training model.

The core concept of whole person education theory is mainly reflected in the comprehensiveness of the training goals, which aim to cultivate people who are both moral and talented, with physically and mentally healthy bodies, servants of society, who are good at innovation [16]. These traits are not only required for the acquisition of knowledge and skills, but also to pay attention to the development of personal ideas and the ability to think independently and form judgements [17]. Combined with the above theory, referring to the ‘Post competency model for clinicians in China’ [18], the ‘Six Major Clinical Core Competence Criteria’ proposed by the Accreditation Council for Graduate Medical Education (ACGME) [19], the Canadian Physician Competence Framework [20], etc., the model of the elements of clinical medical personnel training in the new period was preliminarily determined, which consisted of eight dimensions. They were theoretical medical knowledge, clinical skills practice, public health knowledge, technological innovation capacity, lifelong learning needs, thinking and judgement ability, medical humanistic literacy, and international exchange vision.

### Preliminary scale development

#### General data of respondents

The data in question was self-prepared, and included sex, age, education, type of hospital in which the participants served, whether they were a family doctor, professional title, and length of service.

#### Item development

With the search terms of physician training, post needs, theoretical medical knowledge, clinical skills, clinical practice, public health, scientific research needs, lifelong learning, thinking judgement, humanistic medical education, international communication vision, medical education, physician development, and others, we searched CNKI, Wanfang database, PubMed, Web of science, and other domestic and foreign databases. We then collected and read the literature related to clinician training factors and post needs, screened and drafted item pools, and formed an initial questionnaire with 67 items.

#### Medical theoretical knowledge

Medical theoretical knowledge included basic medical knowledge, which was acquired earlier in the process of medical education; in addition, there was clinical medical theoretical knowledge, integrated knowledge of basic medicine and clinical medicine, and general theoretical knowledge gained after participating in clinical or social practice [21–23]. We also selected the curriculum content of disease prevention and health promotion, knowledge of new fields of medicine, and interdisciplinary knowledge of medical science proposed in the strategic background of ‘Healthy China’ [24].

#### Clinical skills practice

Clinical skills practice referred to the competency index of resident posts proposed by Li Dunqing [25], and the tree model of WONCA (World Organization of National Colleges), which are the acronym comprising the first five initials of the World Organization of National Colleges, Academies and Academic Association of General Practitioners/Family Physicians [26]. It was all combined with the content in the ‘Guidelines for the Diagnosis and Treatment Operations of General Practitioners’ [27], which contained diagnosis, treatment process, and skill operation [28].

#### Public health knowledge

This concerned the study of the countermeasures of the World Health Organization and other countries that faced public health emergencies—such as the COVID-19 pandemic—and their relevant knowledge of public health incidents. This public health knowledge highlighted the paths that help improve the efficiency of public health governance and involved the ability to cope with public health emergencies, ability to solve practical problems, and knowledge of infectious disease prevention [29, 30].

#### Technological innovation capability

This involved the work of Zheng Chuanfen [31], Zhang Lingling [32], and other researchers on the current state of clinicians’ scientific research. Accordingly, technological innovation capability was defined as basic scientific research skills—including literature retrieval, clinical scientific research design, experimental development, data processing—as well as the ability to output scientific research views—such as article writing, project application, and patent application—would affect the development of doctors’ scientific research innovation.

#### Lifelong learning needs

Heather Armson et al. [33] believed that assessing learning needs was a key step in continuing education. Lifelong learning ability combined the knowledge needs of clinical specialists and general practitioners and extracted the humanistic learning content, such as psychology, mental health, and law, that clinicians in China lacked [34]. Moreover, it included community health management abilities such as the treatment, management, and health education of common community diseases [35].

#### Thinking and judgement ability

Referring to Li Zhongyan’s [36] elaboration on the concept and definition of clinical thinking ability by domestic and foreign scholars, thinking and judgement ability summarised and prepared items that could reflect the thinking and judgement ability of doctors in the process of clinical diagnosis and treatment. This included procedures such as establishing auxiliary examinations, offering differential diagnoses, and making clinical decisions in combination with the clinical information of patients.

#### Medical humanistic literacy

Public health emergencies and frequent medical disputes exposed the existing problems in the improvement of humanistic literacy in medical education in China. It was then considered necessary to pay attention to the cultivation of quality, including medical service spirit, doctor-patient communication ability, medical ethics, and medical psychological skills [37–39].

#### International exchange vision

The document, ‘Promoting the Joint Construction of ‘One Belt and One Road’ Education Action’ issued by the Ministry of Education of China in 2017, mentions that universities along the line were encouraged and supported to jointly cultivate medical professionals. This endeavour highlighted the direction for medical education to cultivate medical talents with international vision, such as international knowledge reserve, cross-cultural communication ability, and world vision [40, 41]. Table 1 shows specific scale dimensions and measurement items.

**Table 1:** An initial model of clinician training elements in the new era

Dimensions	Items
A) Medical theoretical knowledge	A1 Relevant theoretical knowledge of basic medicine
	A2 Theoretical knowledge of clinical medicine
	A3 Integrate knowledge of basic medicine and clinical medicine
	A4 Theoretical knowledge of Chinese and Western medicine
	A5 Theoretical knowledge of general medicine
	A6 Disease prevention and health promotion course content
	A7 New medical fields such as artificial intelligence, precision medicine, and translational medicine
	A8 Professional knowledge of the cross-integration of medicine and science, liberal arts, engineering, and other disciplines
B) Clinical skills practice	B1 Take accurate medical history
	B2 Comprehensively and systematically perform a physical examination
	B3 Skillfully and properly conduct basic surgical operations
	B4 Skillfully and properly conduct basic internal medicine operations
	B5 Proficiency in basic operational diagnostic procedures
	B6 Master cardio-pulmonary resuscitation (CPR)
	B7 Community management of chronic diseases
	B8 Master the establishment and management of general medical health records
	B9 Effective communication and standardisation to resolve doctor-patient conflicts
C) Public health knowledge	C1 Master the principles of prevention and control of COVID-19
	C2 Regularly participate in emergency drills for public health emergencies
	C3 Be familiar with the responsibilities of medical staff in the ‘National Emergency Response Plan for Public Health Emergencies’
	C4 Understand the ‘Law of the People’s Republic of China on the Prevention and Treatment of Infectious Diseases’
	C5 Be able to promote new theories, new technologies, new methods and health education for disease prevention and control
	C6 Be able to use digital technologies such as big data and artificial intelligence to solve practical problems such as etiological detection, on-site investigation, and epidemic research and judgement
D) Technological innovation capability	D1 Learn clinical research design
	D2 Basic experimental theory and technology training
	D3 Study of epidemiology and statistical methodology
	D4 Data collection and literature retrieval training
	D5 Data processing method
	D6 Article writing guidelines
	D7 Guidelines for project application
	D8 Guidelines for patent application
	D9 Guidelines for medical ethics
E) Lifelong learning needs	E1 Doctor-patient communication methods and skills
	E2 Medical psychology and mental hygiene
	E3 New clinical progress
	E4 Medical related laws and regulations
	E5 Hospital management knowledge/skills
	E6 Cutting-edge diagnosis and treatment technology skills training
	E7 Healthcare knowledge for special populations
	E8 Rehabilitation medicine related knowledge
	E9 Community health service management
F) Thinking and judgement ability	F1 Be able to analyse the underlying causes of clinical manifestations from different perspectives
	F2 Be able to respond quickly and accurately to the patient’s condition
	F3 Ability to use correct physical examination techniques to obtain and identify various signs
	F4 Sensitive attention to clinical phenomena and subtle changes
	F5 When collecting patient data, different information can be automatically classified in the brain
	F6 Strong clinical thinking
	F7 Reasonable skills in communicating with the patient and the ability to accurately understand the patient
	F8 The ability to translate medical questions you encounter in clinical practice into clear, specific, and answerable questions before implementing measures
	F9 Ability to retrieve information and evidence using evidence-based medicine databases and the Internet
G) Medical humanistic literacy	G1 Principles of optimising diagnosis and examination programmes
	G2 Doctor-patient communication skills
	G3 Everything is patient-centred
	G4 Patient’s right to medical informed consent
	G5 Privacy protection for patients
	G6 Strictly abide by the rules and regulations of diagnosis and treatment
	G7 More detailed screening and assessment work
	G8 Mental health education
	G9 Assess the patients’ mental ability
H) International exchange vision	H1 English communication and expression
	H2 World vision
	H3 Understanding of cultural differences
	H4 Cultural confidence
	H5 Knowledge of international rules and international etiquette
	H6 Knowledge of international cutting-edge medical theories and technologies
	H7 Knowledge of international politics, economics, and culture
	H8 Intercultural communication competence

### Subjects

Before we conducted this study, the design method was approved by the ethics review committee of the school. All the participants were informed of the purpose of the study and volunteered to take part in it. Their personal information was kept anonymous.

We operated according to the principle that the sample size should be greater than five times the number of items in the scale. Therefore, from July 7, 2022, to August 10, 2022, a stratified sampling survey method was used to investigate the clinicians in Chinese medical posts using Sojump, an online crowdsourcing platform in mainland China. This service provides functions equivalent to Amazon Mechanical Turk (MTurk) to receive and send the questionnaire; additionally, the survey scope involved the eastern, central, and western regions of China with certain representativeness. A total of 1,086 questionnaires were distributed, and 1,068 valid questionnaires were received, with an effectivity rate of 98.34%, hence the quantity met the requirements. Among them, the sex distribution was 515 males (48.2%) and 553 females (51.8%). The age distribution was as follows: 335 (31.4%) aged 30 and below; 267 (25%) aged 30 to 35 years old; 205 (19.2%) aged 36 to 40 years old; and 261 (24.4%) aged over 41 years old. From a regional perspective, 915 cases were collected in the eastern region, covering the seven provinces of Shanghai, Zhejiang, Hainan, Jiangsu, Fujian, Guangdong, and Shandong, with 341 hospitals. Subsequently, we collected 91 cases from the central region, and the survey subjects were from five hospitals in the two provinces of Anhui and Jiangxi. A total of 61 cases were collected in the western region; specifically, the data came from nine hospitals in the five provinces of Gansu, Guangxi, Sichuan, Yunnan, and Chongqing. Notably, one copy from Northeast China was collected from Jilin Province. According to the classification of China’s tertiary diagnosis and treatment system, 190 were collected from first-level hospitals such as community health service centres and township health centres; 165 were collected from second-level county hospitals; and 713 were collected from third-level general hospitals. The need indicators were scored using the five-point Likert scale and assigned 1 to 5 points with the following choices: ‘really not needed’, ‘not needed’, ‘fair’, ‘more needed’, and ‘really needed’, respectively. The higher scores indicated that clinical posts had a stronger need for this knowledge. SPSS 26.0 and Amos 24.0 software were used for the statistical analysis of the data.

## Results

### Project analysis

The item analysis of the questionnaire was conducted using the critical ratio (CR) method and the homogeneity test method. The CR method was implemented as follows: the top 27% of the total score of the surveyed subjects were set as the high group, and the bottom 27% as the low group. Two independent samples t-tests were used for the two groups. The items whose critical ratio between the two groups did not reach a significant level (t < 3 or *p* > 0.05, at this time, the t value is the CR value) were deleted. The homogeneity test method was implemented as follows: Pearson’s product-moment correlation method was used to calculate the correlation between the total score of the scale and each item; furthermore, the items with a correlation coefficient *r* < 0.4 were deleted, indicating that those items had a low correlation to the scale theme. Table 2 shows the results.

**Table 2:** Project analysis results

Item	T-test	Pearson’s r	Item	T-test	Pearson’s r
A1	21.336***	0.568***	E3	21.866***	0.773***
A2	16.235***	0.552***	E4	33.559***	0.767***
A3	19.579***	0.547***	E5	36.652***	0.697***
A4	42.654***	0.492***	E6	25.393***	0.752***
A5	34.413***	0.600***	E7	38.045***	0.744***
A6	40.074***	0.617***	E8	36.216***	0.739***
A7	37.599***	0.603***	E9	37.689***	0.722***
A8	39.350***	0.608***	F1	32.142***	0.844***
B1	15.913***	0.689***	F2	28.405***	0.827***
B2	18.710***	0.667***	F3	34.781***	0.839***
B3	21.931***	0.528***	F4	33.829***	0.829***
B4	24.061***	0.666***	F5	34.700***	0.833***
B5	18.469***	0.669***	F6	30.539***	0.817***
B6	14.597***	0.633***	F7	31.716***	0.818***
B7	33.844***	0.611***	F8	39.349***	0.848***
B8	34.718***	0.617***	F9	39.074***	0.846***
B9	18.272***	0.729***	G1	26.280***	0.826***
C1	20.796***	0.751***	G2	24.963***	0.811***
C2	33.983***	0.765***	G3	34.673***	0.773***
C3	34.948***	0.756***	G4	31.747***	0.819***
C4	36.137***	0.770***	G5	30.751***	0.829***
C5	28.120***	0.724***	G6	32.896***	0.836***
C6	27.317***	0.727***	G7	34.494***	0.841***
D1	31.229***	0.714***	G8	31.485***	0.840***
D2	33.530***	0.755***	G9	31.265***	0.839***
D3	36.227***	0.763***	H1	25.511***	0.677***
D4	34.392***	0.763***	H2	30.208***	0.772***
D5	31.897***	0.752***	H3	32.003***	0.767***
D6	37.464***	0.768***	H4	22.589***	0.774***
D7	35.357***	0.764***	H5	45.393***	0.737***
D8	36.655***	0.764***	H6	32.559***	0.755***
D9	39.143***	0.786***	H7	51.059***	0.748***
E1	29.546***	0.729***	H8	47.923***	0.748***
E2	35.090***	0.754***			

^***^ *P* < 0.001 (2-tailed)

As can be seen from Table 2, all the t values of the 67 items exhibited positive and significant relationships (t > 3 and *p* < 0.001); moreover, the correlation coefficient *r* values between each item and the total score ranged between 0.492 and 0.848 (*p* < 0.001). Therefore, all 67 items were retained.

### Construct validity

#### Exploratory factor analysis

**Fig.1:**
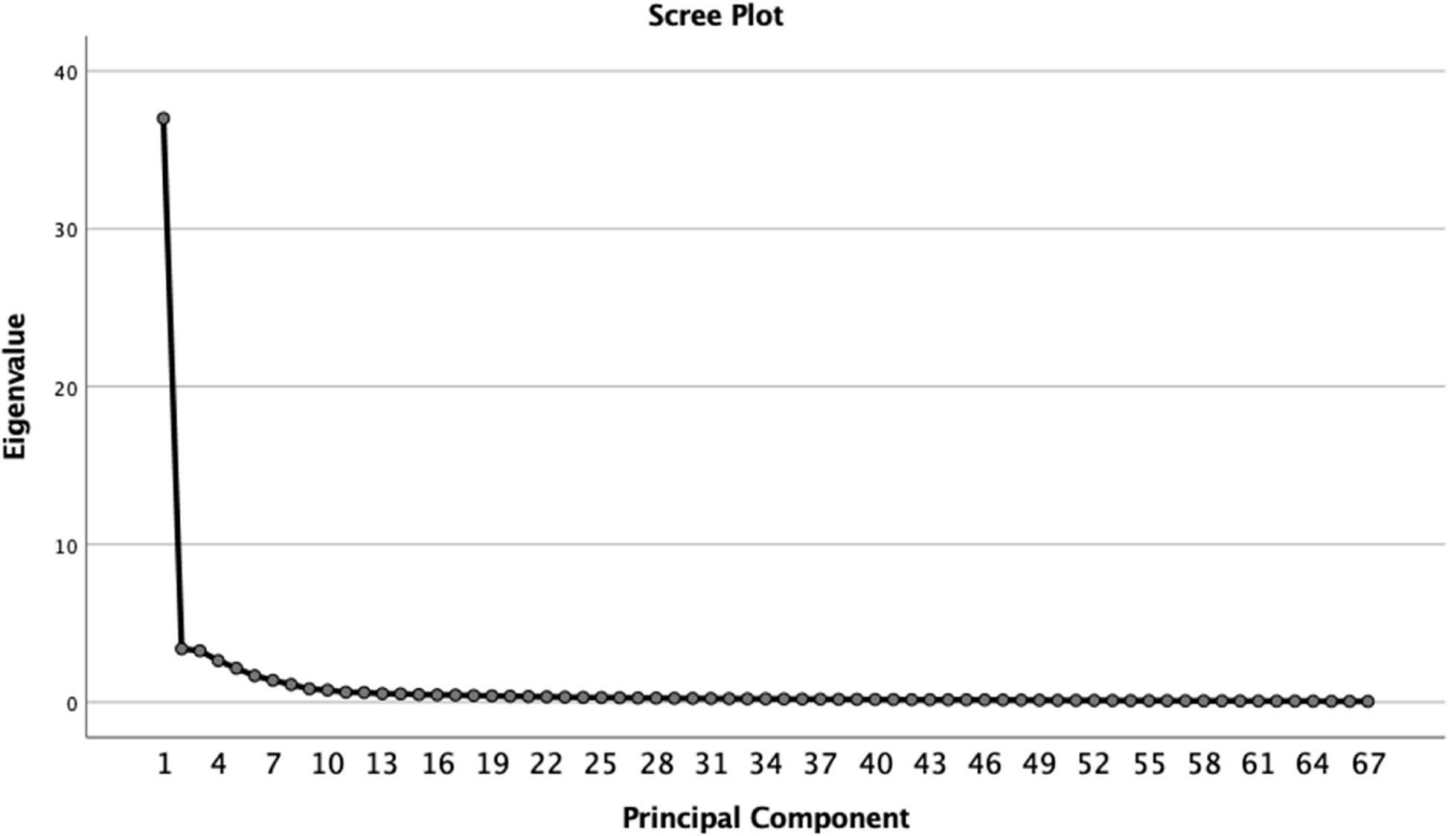
Screen plot

The Kaiser–Meyer–Olkin (KMO) test for sampling adequacy and Bartlett’s Sphericity Test (BST) to measure the homogeneity of variance were assessed on 67 items. The BST was significant (*p* < 0.001) and the KMO value was 0.979, which was above the commonly recommended value of 0.6, indicating that the data were suitable for factor analysis. We conducted a principal components analysis (PCA) with a varimax orthogonal rotation. Based on the existing research [42], items with a lower load factor than 0.4 and a cross load in each dimension were excluded. Specifically, these items were B6, B7, B8, B9, E3, G3, G4, G5, G6, G7, and others in Table 1, and the factor loadings of the remaining 57 entries were all between 0.423 and 1.043. The final PCA analysis revealed the presence of eight PCs with eigenvalues of > 1, thereby explaining 78.542% of the variance (Table 3). In addition, examining the scree plot showed an inflection, which justifies the retention of the eight factors (Fig. 1). The medical theoretical knowledge in Table 1 was divided into two factor models, namely ‘PC 5 interdisciplinary knowledge’ and ‘PC 8 basic clinical knowledge’, according to the factor contribution. Thinking and judgement ability and medical humanistic literacy were combined into one factor model, which integrated thinking and judgement ability into medical humanistic literacy. Moreover, PC 6 was renamed ‘clinical skill operation’ because of its content.

**Table 3:** Construct validity by principal component analysis using varimax rotation

Items	PC 1	PC 2	PC 3	PC 4	PC 5	PC 6	PC 7	PC 8
	Medical humanistic literacy	Technological innovation capability	International exchange vision	Lifelong learning needs	Interdisciplinary knowledge	Clinical skill operation	Public health knowledge	Basic clinical knowledge
A1 Relevant theoretical knowledge of basic medicine								.881
A2 Theoretical knowledge of clinical medicine								.838
A3 Integrate knowledge of basic medicine and clinical medicine								.844
A4 Theoretical knowledge of Chinese and Western medicine					.641			
A5 Theoretical knowledge of general medicine					.586			
A6 Disease prevention and health promotion course content					.791			
A7 New medical fields such as artificial intelligence, precision medicine, and translational medicine					.909			
A8 Professional knowledge of the cross-integration of medicine and science, liberal arts, engineering, and other disciplines					.898			
B1 Take accurate medical history						.720		
B2 Comprehensively and systematically perform a physical examination						.843		
B3 Skillfully and properly conduct basic surgical operations						.704		
B4 Skillfully and properly conduct basic internal medicine operations						.878		
B5 Proficiency in basic operational diagnostic procedures						.757		
C1 Master the principles of prevention and control of COVID-19							.658	
C2 Regularly participate in emergency drills for public health emergencies							.950	
C3 Be familiar with the responsibilities of medical staff in the ‘National Emergency Response Plan for Public Health Emergencies’							1.019	
C4 Understand the ‘Law of the People’s Republic of China on the Prevention and Treatment of Infectious Diseases’							.837	
C5 Be able to promote new theories, new technologies, new methods and health education for disease prevention and control							.581	
C6 Be able to use digital technologies such as big data and artificial intelligence to solve practical problems such as etiological detection, on-site investigation, and epidemic research and judgement							.580	
D1 Learn clinical research design		.907						
D2 Basic experimental theory and technology training		.766						
D3 Study of epidemiology and statistical methodology		.876						
D4 Data collection and literature retrieval training		.869						
D5 Data processing method		.964						
D6 Article writing guidelines		.925						
D7 Guidelines for project application		.970						
D8 Guidelines for patent application		.826						
D9 Guidelines for medical ethics		.749						
E1 Doctor-patient communication methods and skills				.712				
E2 Medical psychology and mental hygiene				.780				
E4 Medical related laws and regulations				.708				
E5 Hospital management knowledge/skills				.854				
E6 Cutting-edge diagnosis and treatment technology skills training				.423				
E7 Healthcare knowledge for special populations				.917				
E8 Rehabilitation medicine related knowledge				.950				
E9 Community health service management				.980				
F1 Be able to analyse the underlying causes of clinical manifestations from different perspectives	.886							
F2 Be able to respond quickly and accurately to the patient’s condition	.913							
F3 Ability to use correct physical examination techniques to obtain and identify various signs	.915							
F4 Sensitive attention to clinical phenomena and subtle changes	1.043							
F5 When collecting patient data, different information can be automatically classified in the brain	.970							
F6 Strong clinical thinking	.971							
F7 Reasonable skills in communicating with the patient and the ability to accurately understand the patient	.923							
F8 The ability to translate medical questions you encounter in clinical practice into clear, specific, and answerable questions before implementing measures	.951							
F9 Ability to retrieve information and evidence using evidence-based medicine databases and the Internet	.808							
G1 Principles of optimising diagnosis and examination programmes	.707							
G2 Doctor-patient communication skills	.733							
G8 Mental health education	.502							
G9 Assess the patients’ mental ability	.599							
H1 English communication and expression			.764					
H2 World vision			.711					
H3 Understanding of cultural differences			.755					
H4 Cultural confidence			.623					
H5 Knowledge of international rules and international etiquette			.973					
H6 Knowledge of international cutting-edge medical theories and technologies			.900					
H7 Knowledge of international politics, economics, and culture			.962					
H8 Intercultural communication competence			.966

#### Confirmatory factor analysis

The fitting degree of the above model was verified in Table 3 according to the following eight latent variables: medical humanistic literacy, technological innovation capability, international exchange vision, lifelong learning needs, interdisciplinary knowledge, clinical skill operation, public health knowledge, and basic clinical knowledge. The results showed that the five indicators of Chi-squared degrees of freedom ratio (χ2/df), comparative fit index (CFI), Tucker-Lewis index (TLI), normative fit index (NFI) and incremental fit index (IFI) did not meet the reference value requirements.

**Fig. 2:**
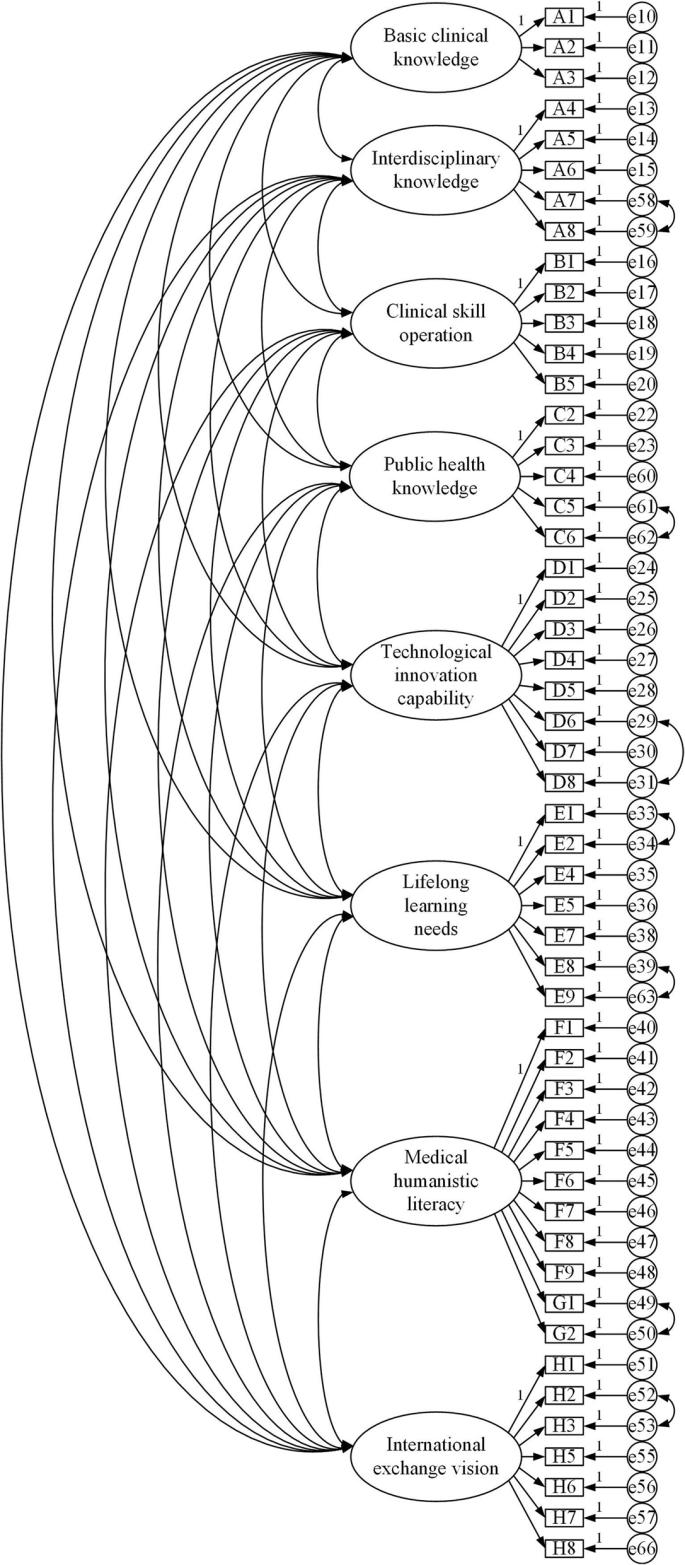
Fitted path diagram after correction

The initial model was revised according to the modification indices (MI). The entries C1, D9, E6, G8, G9 and H4 that were strongly correlated with multiple dimensions were eliminated, and the residual items e58 and e59, e61 and e62, e29 and e31, e33 and e34, e39 and e63, e49 and e50, and e52 and e53 were established. After the correction, the fitting indices all reached the model fitting standard [42]. The fit of the revised model was better. Table 4 presents the revised culture element model, Table 5 shows the specific indicators of model fitting, and Fig. 2 illustrates the revised fitting path diagram.

**Table 4:** A revised model of clinician training elements in the new era

Dimensions	Items
Basic clinical knowledge	A1 Relevant theoretical knowledge of basic medicine
	A2 Theoretical knowledge of clinical medicine
	A3 Integrate knowledge of basic medicine and clinical medicine
Interdisciplinary knowledge	A4 Theoretical knowledge of Chinese and Western medicine
	A5 Theoretical knowledge of general medicine
	A6 Disease prevention and health promotion course content
	A7 New medical fields such as artificial intelligence, precision medicine, and translational medicine
	A8 Professional knowledge of the cross-integration of medicine and science, liberal arts, engineering, and other disciplines
Clinical skill operation	B1 Take accurate medical history
	B2 Comprehensively and systematically perform a physical examination
	B3 Skillfully and properly conduct basic surgical operations
	B4 Skillfully and properly conduct basic internal medicine operations
	B5 Proficiency in basic operational diagnostic procedures
Public health knowledge	C2 Regularly participate in emergency drills for public health emergencies
	C3 Be familiar with the responsibilities of medical staff in the ‘National Emergency Response Plan for Public Health Emergencies’
	C4 Understand the ‘Law of the People’s Republic of China on the Prevention and Treatment of Infectious Diseases’
	C5 Be able to promote new theories, new technologies, new methods and health education for disease prevention and control
	C6 Be able to use digital technologies such as big data and artificial intelligence to solve practical problems such as etiological detection, on-site investigation, and epidemic research and judgement
Technological innovation capability	D1 Learn clinical research design
	D2 Basic experimental theory and technology training
	D3 Study of epidemiology and statistical methodology
	D4 Data collection and literature retrieval training
	D5 Data processing method
	D6 Article writing guidelines
	D7 Guidelines for project application
	D8 Guidelines for patent application
Lifelong learning needs	E1 Doctor-patient communication methods and skills
	E2 Medical psychology and mental hygiene
	E4 Medical related laws and regulations
	E5 Hospital management knowledge/skills
	E7 Healthcare knowledge for special populations
	E8 Rehabilitation medicine related knowledge
	E9 Community health service management
Medical humanistic literacy	F1 Be able to analyse the underlying causes of clinical manifestations from different perspectives
	F2 Be able to respond quickly and accurately to the patient’s condition
	F3 Ability to use correct physical examination techniques to obtain and identify various signs
	F4 Sensitive attention to clinical phenomena and subtle changes
	F5 When collecting patient data, different information can be automatically classified in the brain
	F6 Strong clinical thinking
	F7 Reasonable skills in communicating with the patient and the ability to accurately understand the patient
	F8 The ability to translate medical questions you encounter in clinical practice into clear, specific, and answerable questions before implementing measures
	F9 Ability to retrieve information and evidence using evidence-based medicine databases and the Internet
	G1 Principles of optimising diagnosis and examination programmes
	G2 Doctor-patient communication skills
International exchange vision	H1 English communication and expression
	H2 World vision
	H3 Understanding of cultural differences
	H5 Knowledge of international rules and international etiquette
	H6 Knowledge of international cutting-edge medical theories and technologies
	H7 Knowledge of international politics, economics, and culture
	H8 Intercultural communication competence

**Table 5:** Confirmatory factor analysis fitting results

Fit indices	Fit criteria or thresholds	The initial model	The revised model
Chi-squared degrees of freedom ratio (χ^2^/df)	< 3 means ideal, 3 ~ 5 represents acceptable	6.914	4.854
RMSEA	< 0.08	0.074	0.060
SRMR	< 0.05	0.048	0.041
CFI	> 0.90	0.887	0.934
TLI	> 0.90	0.881	0.929
NFI	> 0.90	0.870	0.919
IFI	> 0.90	0.887	0.934

*RMSEA* Root mean squared error of approximation, *SRMR* Standardised root mean square residual, *CFI* Comparative fit index, *TLI* Tucker-Lewis index, *NFI* Normative fit index, *IFI* Incremental fit index

### Convergent validity

Table 6 shows that the average variance extraction (AVE) of each dimension was greater than 0.5. Furthermore, the combined reliability was greater than 0.6, indicating that the convergent validity of the entire scale was obvious.

**Table 6:** AVE and combined reliability values of each dimension of the scale

Dimensions	AVE	Combined reliability
Basic clinical knowledge	0.763	0.906
Interdisciplinary knowledge	0.655	0.904
Clinical skill operation	0.677	0.908
Public health knowledge	0.753	0.938
Technological innovation capability	0.819	0.973
Lifelong learning needs	0.739	0.952
Medical humanistic literacy	0.822	0.981
International exchange vision	0.804	0.966

*AVE* Average variance extraction

### Discriminant validity

As shown in Table 7, there was a significant correlation between basic clinical knowledge, interdisciplinary knowledge, clinical skill operation, public health knowledge, technological innovation capability, lifelong learning needs, medical humanistic literacy, and international exchange vision (*p* < 0.001). Most of the correlation coefficients between variables were in the range of 0.1 to 0.7, and all were less than the square root of AVE, suggesting good discriminant validity.

**Table 7:** Discriminant validity of training elements scale

Dimensions	Basic clinical knowledge	Interdisciplinary knowledge	Clinical skill operation	Public health knowledge	Technological innovation capability	Lifelong learning needs	Medical humanistic literacy	International exchange vision
Basic clinical knowledge	0.763							
Interdisciplinary knowledge	0.659***	0.655						
Clinical skill operation	0.630***	0.523***	0.677					
Public health knowledge	0.543***	0.593***	0.651***	0.753				
Technological innovation capability	0.465***	0.491***	0.613***	0.661***	0.819			
Lifelong learning needs	0.410***	0.609***	0.555***	0.699***	0.628***	0.739		
Medical humanistic literacy	0.554***	0.525***	0.723***	0.729***	0.729***	0.697***	0.822	
International exchange vision	0.364***	0.510***	0.519***	0.605***	0.621***	0.674***	0.701***	0.804
Square root of AVE	0.873	0.809	0.823	0.868	0.905	0.860	0.907	0.897

^***^ *P* < 0.001 (2-tailed), The diagonal line is the average variance extraction amount of the average variance extraction (AVE) evaluation

### Reliability

All measures of the two types of internal consistency reliability were satisfactory for each dimension of the revised Training Elements Scale (Table 8). The Cronbach’s alpha coefficient of the full scale was 0.981, and the split-half reliability was 0.903. The minimum value of the total reliability and the reliability indicators of each dimension were all greater than 0.8. These observations showed that the scale had good internal consistency, stability, and reliability.

**Table 8:** Reliability

Dimensions	Cronbach’s alpha coefficient	Split-half reliability
Basic clinical knowledge	0.903	0.822
Interdisciplinary knowledge	0.906	0.839
Clinical skill operation	0.900	0.884
Public health knowledge	0.942	0.835
Technological innovation capability	0.973	0.948
Lifelong learning needs	0.954	0.903
Medical humanistic literacy	0.981	0.957
International exchange vision	0.968	0.908
Full Scale	0.981	0.903

## Discussion

### Scale items reflect the requirements of the times

This research was conducted under the background of the new era and a new trend of demand for high-quality and diversified development of medical education in the post-pandemic period, characterised by the Chinese government attaching great importance to public health, as well as promoting the implementation of its ‘Healthy China’ strategy [43]. Reviewing the relevant literature, we found that researchers in the new era are behind in presenting research on constructing a model of training elements to guide medical education in colleges and universities based on the needs of clinicians. In contrast, the research on constructing a job competency model is relatively abundant. Presently, there are the Huang Qixiang’s clinical resident competency model, Zhang Xiujun’s clinician’s post competency model, Huang Xiaoling’s excellent medical talent competency model, Professor Sun Baozhi’s general model of clinician job competence, Ronald M. Epstein’s competency model, and others [44]. Compared with the above studies, the model constructed in this study takes the job requirements of clinicians as the starting point, and establishes the internal linkage of doctors, colleges, and students, which helps to provide more specific target directions for medical education reform and student learning in colleges and universities. The preparation of the scale is guided by important documents such as the Chinese government’s ‘Opinions on Strengthening the Collaboration between Medicine and Education to Implement the Excellent Doctor Education and Training Program 2.0’ and ‘Guiding Opinions on Accelerating the Innovative Development of Medical Education’ [2, 7]. Among them, the dimension of medical humanistic literacy is a reflection on the current situation of ‘emphasising theory and ignoring humanities’ in medical education in China. It is also a manifestation of the lack of medical humanities in clinical cures exposed in the fight against the COVID-19 pandemic. The development of medicine contains the nourishment and cultivation of the humanistic spirit. Given this, strengthening humanistic medical education is the historical mission and important responsibility of contemporary medical educators [45]. Additionally, original clinical thinking in the dimension of medical humanistic literacy emphasises the integration of humanistic care into the whole process of clinical diagnosis and treatment, which contributes to improving the quality of medical services and highlights the humanistic characteristics of medicine, not just the treatment of the disease itself. There is an emergence of new specialties, such as materials medicine, smart medicine, and precision medicine, that exist within the framework of the intersection between medicine and big data, medicine and humanities and social sciences, and medicine and artificial intelligence [43]. This connection shows that the knowledge content of interdisciplinary knowledge in the new era needs to be strongly emphasised in the teaching of medical students. Therefore, the original theoretical medical knowledge dimension is divided into two dimensions: clinical basic knowledge and interdisciplinary knowledge.

### The dimensions of the scale model are comprehensive and extensive

The scale builds a model of clinician training elements in the new era, covering the eight dimensions, including basic clinical knowledge, interdisciplinary knowledge, clinical skill operation, public health knowledge, technological innovation capability, lifelong learning needs, medical humanistic literacy, and international exchange vision. Basic clinical knowledge is the focus of the entire medical undergraduate education as well as the starting point for the cultivation of clinical thinking ability [22]. Interdisciplinary knowledge is the product of the era in which medical education actively adapts to the new scientific and technological revolution and the new industrial revolution, and is the basis for the application of cutting-edge clinical diagnosis and treatment technologies, diagnosis and treatment decisions, and health management [43]. Clinical medicine is characterised by strong practice and strong skills, which requires medical students to be proficient in clinical skills operation [46]. Public health knowledge is a weak link in the current ‘big health’ and ‘full cycle’ medical training system. Therefore, strengthening the integration and application of public health knowledge will help reconstruct the public health system and improve the structure of medical personnel training [47]. The strategic position of medical science and technology is becoming increasingly prominent in health maintenance, and cultivating medical students’ scientific and technological innovation abilities is the reserve force for the implementation of the ‘Healthy China’ strategy [48]. As a profession of continuous development, doctors should be committed to the concept of lifelong learning and propose requirements for the culture and curriculum of lifelong learning [49]. The combination of medical humanistic literacy and medical science practice is an urgent need to achieve the goal of humanised medical care [50]. Hence, improving medical students’ vision of international exchanges is of great benefit to promoting the common development of medicine worldwide, broadening the field of international exchanges, and enhancing the international competitiveness of China’s medical education system [51, 52]. The model constructed in this study, from college education to post-graduation education and continuing education, from the professional knowledge of the first classroom to the humanistic literacy of the second classroom, to the expansion of international vision, fully integrates all elements of a medical student’s growth, indicating that the scale can comprehensively reflect training elements of clinicians.

### The research scope is representative of the population

The reform of tertiary medical institutions has been implemented in China to reasonably allocate medical resources and alleviate the difficulty of seeking medical treatment in large hospitals. The township health centres and community hospitals are classified as first-class hospitals, which are mainly responsible for the prevention, medical treatment, and health and rehabilitation services in certain areas. The county hospitals are classified as secondary hospitals, which mainly undertake multiple community comprehensive medical and health services and certain teaching and scientific research tasks. A general hospital with a tertiary class A designation is classified as a tertiary hospital, mainly providing high-level specialised medical and health services and higher education teaching and scientific research tasks [53]. In this way, a whole-process, seamless health management system has been constructed so patients can seek reasonable medical and health services at a proper medical level at a reasonable medical and health institution [54].

Based on China’s new reform background, we conducted surveys of clinicians in tertiary medical institutions, reflecting the comprehensiveness and representativeness of Chinese doctors’ job requirements. The goal of the training of medical skills in colleges and universities is to transport these abilities to the whole society, especially in the undergraduate stage of clinical medicine. Furthermore, it is particularly important to comprehensively investigate the needs of different medical institutions for the training of clinicians in order to conduct the establishment of a solid foundation, wide calibre, and strong practice.

### Practical application analysis of scale model

The scale model can be used in various ways. First, in the educational stage of colleges and universities. In revising the training program for clinical medicine professionals, medical colleges and universities can refer to the capacity training needs of clinical doctors in the new era and integrate the eight dimensions into the training programmes. For example, in earning content for new medical fields such as artificial intelligence, precision medicine and other interdisciplinary knowledge, it can guide colleges and universities to set up "Introduction to Artificial Intelligence and Big Data", "Intelligent Analysis of Medical image" and similar courses. To cultivate scientific and technological innovation ability, it can guide colleges and universities to use the second classroom to carry out students' scientific research design and project application training and other content. Since college education offers comprehensive cultivation of students' knowledge and ability, the content of the scale needs to run through the whole process of undergraduate clinical medicine education. Secondly, it is useful in the post-graduation education stage. Doctors at this stage have been engaged in clinical work, and the training content required by doctors at different levels of medical institutions has different emphases. Therefore, doctors in clinical institutions can score the needs of the scale of training factors according to their own needs. For example, we can set 1–5 points for each option of the scale or set "Yes or No" options. 1–5 points indicate the intensity of the training demand content intention, and the training content can be set according to the intensity of the demand of each dimension. We can also directly choose "Yes or No" options. Training content is also set according to the needs of doctors at different levels of medical and health institutions. Finally, after doctors are trained, they can also self-test and score the the eight dimensions, and conduct self-assessment according to their mastery of the training content. For those who think that the training content has not reached the desired level of training, they can arrange further training. Such demand-oriented training education can also stimulate the doctors’ learning autonomy. You can examine your lack of knowledge in clinical work on the content of the training scale, and regularly participate in all kinds of relevant knowledge training.

## Conclusion

The Scale of Factors for Training Clinicians in the New Era has good reliability and validity, and the survey results can be used as reference for the training of clinicians in China in the new era. The questionnaire is comprehensive and easy to understand. It can be widely used in medical colleges and universities as a reference for reforming medical training content. It has significance for the reconstruction of theoretical teaching content and the strengthening of practical teaching projects. In particular, it provides a new ddirection for medical students in terms of increasing the weight of humanistic spirit training and expanding their global vision. It can also be widely used in the continued education of clinicians after graduation. This could make up for the lack of knowledge of doctors in the process of clinical work, improve the content of continuing education of doctors, and provide a reference for doctors' lifelong learning content.

## Data Availability

Data and materials can be obtained from the corresponding author upon request.

## Abbreviations

COVID-19: Coronavirus disease 2019
ACGME: Accreditation Council for Graduate Medical Education
WONCA: World Organization of National Colleges
MTurk: Mechanical Turk
CR: Critical ratio
KMO: Kaiser–Meyer–Olkin
BST: Bartlett’s sphericity test
PCA: Principal components analysis
RMSEA: Root mean squared error of approximation
SRMR: Standardised root mean square residual
CFI: Comparative fit index
TLI: Tucker-Lewis index
NFI: Normative fit index
IFI: Incremental fit index
MI: Modification indices
AVE: Average variance extraction
